# The LiaSR Two-Component System Regulates Resistance to Chlorhexidine in *Streptococcus mutans*

**DOI:** 10.3390/microorganisms12030468

**Published:** 2024-02-26

**Authors:** Shan Huang, Jing Huang, Jingyun Du, Yijun Li, Minjing Wu, Shuai Chen, Ling Zhan, Xiaojing Huang

**Affiliations:** 1 Fujian Key Laboratory of Oral Diseases, Fujian Provincial Engineering Research Center of Oral Biomaterial, Stomatological Key Lab of Fujian College and University, School and Hospital of Stomatology, Fujian Medical University, 246 Yangqiao Zhong Road, Fuzhou 350002, China; shanhuangfjmu@163.com (S.H.); huangjing56@163.com (J.H.); dujingyun2003@163.com (J.D.); liyijun1923@163.com (Y.L.); minjingwu@foxmail.com (M.W.); drchenshuai@163.com (S.C.); 2 Department of Stomatology, Zhongshan Hospital Affiliated to Xiamen University, Xiamen 361004, China; 3 Division of Pediatric Dentistry, Department of Orofacial Sciences, University of California, San Francisco, CA 94143, USA

**Keywords:** *Streptococcus mutans*, LiaSR two-component system, chlorhexidine resistance, efflux pump, cells envelope stress responses

## Abstract

Chlorhexidine (CHX) is widely considered to be the gold standard for preventing dental caries. However, it is possible to induce resistance to CHX. The LiaSR two-component system has been identified that contributed to CHX resistance in *Streptococcus mutans*, which is one of the major pathogens in dental caries. However, the underlying mechanisms remain unclear. In this study, an MIC assay and a viability assessment demonstrated that after deleting the *liaS* and *liaR* genes, the sensitivity of mutants could increase. The Nile Red efflux assay exhibited that the efflux rates of mutants were significantly decreased. The RT-qPCR results indicated that the LiaSR two-component system-mediating influence on the expression of *lmrB* in *S. mutans* contributed to the efflux rate. The hydrophobicity assay and membrane potential assay showed that the mutants had higher levels of hydrophobicity and depolarization, suggesting that their membranes were more easily disturbed. The TEM graphs revealed that the border of the cell membrane was unclear in mutants compared with the wild-type strain, indicating that the cell envelope’s stress response may have been inhibited. While the surface charge of mutants showed no significant difference in the wild-type strain according to the result of cytochrome c-based charged determination. This study provides valuable novel insights into the mechanisms of the LiaSR two-component system in the CHX resistance of *S. mutans*.

## 1. Introduction

Controlling microbial biofilm infections is a global challenge [1,2]. This is partly because the bacteria are embedded in the extracellular matrix, which can provide bacteria with a protective environment and promote the bacteria tightly adhering to the interface [3,4]. Dental caries is one of the most prevalent oral infectious diseases. The accumulation of biofilms on teeth can lead to the destruction of dental tissue. *Streptococcus mutans* (*S. mutans*) is one of the etiological agents of dental caries due to its strong capacity for the synthesis of exopolysaccharides, acid production and acid tolerance [5,6,7].

In clinical dental practice, mechanical methods are not sufficient for the removal of biofilms. Therefore, chlorhexidine (CHX) is widely applied because of its broad-spectrum antibacterial effect. CHX is a bis-biguanide agent that carries a positive charge and can be attracted to the negatively charged cell surface, resulting in membrane disruption and leakage of the cell’s contents [8]. It is considered to be the gold standard for dental caries prevention [8].

Although CHX is considered relatively safe, its side effects cannot be ignored, particularly with regard to resistance. Studies have confirmed that continuous exposure to CHX can induce CHX resistance in *S. mutans* [9,10]. In recent years, researchers have focused on developing CHX-incorporated materials to prevent dental caries [11,12]. The release of CHX molecules may increase the exposure time of *S. mutans*, leading to CHX resistance. The emergence of CHX-resistant *S. mutans* has presented new challenges for dental caries treatment and prevention because the cariogenicity of CHX-resistant *S. mutans* appears to be similar to that of the wild-type strains [9], indicating that even under CHX pressure on *S. mutans*, resistant strains can still play an important role in dental caries. However, the related mechanisms of CHX resistance remain unclear, which is crucial for the development of CHX-incorporated materials.

The LiaSR two-component system in *S. mutans* comprises a membrane-bound histidine kinase receptor (LiaS) and a cognate response regulator (LiaR) [13,14,15]. When the LiaS senses the environmental changes, it undergoes autophosphorylation and then transfers the phosphoryl group to the LiaR, altering the LiaR’s affinity to bind to the promoter regions of the target genes and regulate their expression [16]. When the stress disappears, the LiaF can “switch off” this system [16]. The LiaSR two-component system in this study was found to be homologous to the LiaSR two-component system in *Bacillus subtilis* (*B. subtilis*) [17] and the VraSR two-component system in *Staphylococcus aureus* (*S. aureus*) [18], which has been linked to antiseptic resistance. In *S. aureus*, the VraSR two-component system can be activated by CHX to modulate cell envelope stress responses (CESRs), contributing to vancomycin resistance [19]. The LiaSR two-component system plays an important role in CESRs in *B. subtilis* [17]. In *S. mutans*, the deletion of *liaS* and *liaR* may reduce the sensitivity to various antiseptics, including bacitracin and CHX [16,20]. The phenomenon implied that the LiaSR two-component system was associated with CHX-sensitivity in *S. mutans*. However, the underlying mechanisms remain unclear. Therefore, this study aimed to explore whether the LiaSR two-component system is involved in CHX sensitivity in *S. mutans* and to elaborate on the underlying mechanisms. Based on the antimicrobial mechanisms of CHX, bacteria could activate CESRs [19] and change the surface charge to resist CHX [9]. These mechanisms, combined with the fact that CHX can be detoxified by efflux pumps, have led to the hypothesis that the LiaSR two-component system confers CHX resistance on *S. mutans* by mediating its efflux rates, CESRs and surface charge.

## 2. Methods

### 2.1. Bacterial Strains and Culture Conditions

All bacterial strains used in this study are listed in Table 1. *S. mutans* 593 (serotype C), which was isolated from a caries-active adult (number of decayed and filled teeth (DFT) = 10, no missing teeth, and three non-restored cavities) and has been proven to possess high cariogenicity [21]. *S. mutans* 593 liaS^−^ and *S. mutans* 593 liaR^−^ were constructed in a previous study and were defined as *liaS* and *liaR* deletion mutants [22]. *S. mutans* strains were grown in brain–heart infusion (BHI) broth or BHI agar plates under anerobic conditions (37 °C, 80% N_2_, 10% CO_2_, 10% H_2_) in an anerobic incubator (DG250, Don Whitley Scientific, Shipley, UK).

### 2.2. Minimum Inhibitory Concentration (MIC) Assay

A minimum inhibitory concentration assay was performed as previously described [9]. Briefly, CHX was dissolved in BHI broth before serial dilution. Overnight cultures of *S. mutans* were adjusted to 1 × 10^6^ CFU/mL, then 100 μL of a bacterial suspension was used to inoculate the same volume of BHI broth containing the CHX. BHI broth without bacteria was used as the control. The 96-well microplates were incubated at 37 °C for 24 h, and the lowest concentration that inhibited visible bacterial growth was defined as the MIC.

### 2.3. Biofilm Formation and Viability Assessment

Construction of the biofilm was performed in accordance with the method previously described [22]. Mid-exponential phase cells were adjusted to 1 × 10^8^ CFU/mL with BHI broth. Then the bacterial suspension was diluted (1:20) using the BHI broth supplemented with 1% sucrose before being plated into 96-well microplates. The plates were cultured anaerobically at 37 °C.

After 24 h of incubation, the biofilms were rinsed with phosphate-buffered saline (PBS) to remove the planktonic cells. The biofilms were then treated with 0.2% CHX for 0, 15 and 30 min. After treatment, the biofilms were collected using PBS and serially diluted in a PBS solution. The samples were inoculated onto BHI agar plates and incubated for 48 h. Finally, the number of colonies were calculated to analyze the disinfection effect.

### 2.4. Nile Red Efflux Assay

Nile Red is a fluorescent marker whose polarity is similar to that of many antiseptics [23]. Based on the fact that Nile Red is weakly fluorescent in aqueous solutions but strongly fluoresces in a non-polar environment, the variation in fluorescence can be monitored when it enters and exits bacterial cells [24]. The Nile Red efflux assay was conducted according to a protocol published earlier [25], with slight modifications. The construction of *S. mutans* biofilms was performed as previously described. A 20 mM potassium phosphate buffer (pH = 7.0) containing 1 mM MgCl_2_ (PPB) was used to remove the planktonic cells of the samples before being collected with PPB. After centrifugation (4000× *g* for 5 min; Eppendorf, Hamburger, Germany), the cells were pelleted and then resuspended in PPB to an absorbance at 600 nm of 1.0. Carbonyl cyanide m-chlorophenylhydrazone was added to a final concentration of 5 mM. After 15 min, Nile Red was added to a final concentration of 5 μM and incubated on a shaker (140 rpm; 37 °C; NRT-100B, Shanghai, China) in the dark. After incubation, the cells were pelleted by centrifugating (4000× *g* for 5 min; Eppendorf, Hamburger, Germany) before resuspension. Next, 200 μL of the solution was transferred to a 96-well plate. Fluorescence intensity of each well was measured at an excitation wavelength of 525 nm and an emission wavelength of 636 nm using a SpectraMax iD3 Multi-Mode Microplate Reader (Molecular devices iD3, San Jose, CA, USA) for 200 s. Nile Red was triggered by rapid energization with 1 M glucose and monitored for another 820 s.

### 2.5. Transmission Electron Microscopy (TEM) Observation

TEM observation was performed as previously described [9]. Exponential-phase cells were fixed with 2.5% glutaraldehyde overnight. The specimens were prepared for examination. The bacterial ultrastructure was photographed using TEM at magnifications ranging from ×2.5 k to ×50 k at 80 kV.

### 2.6. Membrane Potential Assay

The biofilm was constructed as described above. After incubation, deionized water was used to rinse the biofilm. The biofilm was then incubated in 200 μL deionized water containing 10 μM Bis-(1,3-dibutylbarbituric acid) trimethine oxonol (DiBAC4(3)) in the dark for 30 min. After that, the dye was discarded, and the biofilm was rinsed twice with deionized water. The biofilm was then treated with 0.2% CHX for 15 min. Thereafter, the CHX was discarded. PBS was used to wash the biofilm to remove residual CHX and resuspend the cells. Aliquots of the suspension were serially diluted and were spread on the BHI agar. BHI agar was incubated at 37 °C before counting. The remaining suspension was transferred into a new dark 96-well plate for measurement of the fluorescence. The fluorescence intensity was measured with the SpectraMax iD3 Multi-Mode Microplate Reader (Molecular Devices iD3, San Jose, CA, USA) (λ_ex_: 493 nm and λ_em_: 516 nm).

### 2.7. Assay for Hydrophobicity

To measure the hydrophobicity, a modified protocol was used [9]. Briefly, the overnight culture was pelleted by centrifugation. A phosphate–urea–magnesium sulfate (PUM) buffer was used to resuspend the cells, and the initial optical density at 600 nm was recorded as OD_600 Before_. Next, 5 mL of the bacterial suspension was mixed vigorously with 500 μL n-hexadecane for 30 s before incubation at room temperature for 10 min. After incubation, the OD_600_ of the lower aqueous phase was measured and recorded as OD_600 After_. Hydrophobicity was calculated as follows: (OD_600 Before_ − OD_600 After_)/OD_600 Before_ × 100%.

### 2.8. Cytochrome C-Based Charge Determination of Biofilm-Grown Cells

Construction of the biofilm was conducted as previously described. To measure the charge of the biofilm-grown cells, a modified protocol described by Martin et al. was performed [26]. For this, 3-(*N*-morpholino)propanesulfonic acid (20 mM MOPS, pH = 7.0) was used to harvest the biofilm-grown cells, and the cell suspension was adjusted to OD_600_ = 1.0. Cytochrome c was added to a final concentration of 0.5 mg/mL. Thereafter, the samples were incubated for 15 min at room temperature and were then centrifuged. The amount of cytochrome c remaining in the supernatant was measured by the absorbance at 530 nm.

### 2.9. RNA Isolation and Reverse-Transcription Quantitative PCR (RT-qPCR)

Total RNA was extracted from the samples according to the manufacturer’s instructions (Qiagen, Valencia, CA, USA). The purity and quantity of the RNA were evaluated by NanoDrop 2000 (Thermo Scientific, Waltham, Massachusetts, USA) according to the A_260_/A_280_ ratio. Agarose gel electrophoresis was used to validate the integrity of the RNA. To remove DNA contamination and reverse transcription, the kit from Takara Bio (Otsu, Japan) was used according to the manufacturer’s instructions. RT-qPCR was conducted using SYBR^®^ Premix Ex Taq^TM^ (Tli RNaseH Plus) (Takara, Beijing, China) in a LightCycler 480 quantitative PCR instrument. The primers specific to the target genes are listed in Table 2. The genes’ expression levels were calculated by the normalizing target genes to the respective reference genes on the basis of the 2^−ΔΔCt^ method.

### 2.10. Statistical Analysis

Each experiment was carried out in triplicate. Data were statistically analyzed using the Statistical Package for Social Sciences (SPSS, Inc., Chicago, IL, USA), version 21.0. One-way ANOVA was used to detect the significant effects of the variables. Data were considered significantly different when the two-tailed *p*-value was <0.05.

## 3. Results

### 3.1. Sensitivity of S. mutans to CHX

**Figure 1:**
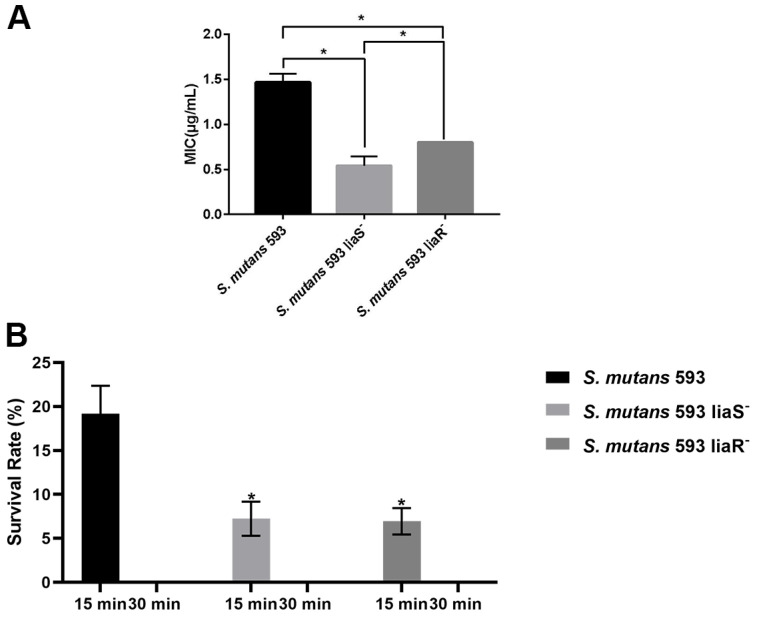
The sensitivity of *S. mutans* to CHX. (**A**) The MICs of *S. mutans*. Values are presented as the mean ± SD. *, *p* < 0.05. (**B**) The survival rates of *S. mutans* after 15 min and 30 min of CHX treatment. *, *p* < 0.05 compared with *S. mutans* 593.

As shown in Figure 1A, the MIC of the wild-type strain was significantly higher than that of the *liaS* and *liaR* deletion mutants. For the biofilm, significant decreases were observed in bacterial number of the *liaS* and *liaR* deletion mutants after 15 min of treatment with 0.2% CHX when compared with the wild-type strain. After 30 min of treatment, no viable cells were observed in the *S. mutans* biofilm (Figure 1B).

### 3.2. Efflux Activity of S. mutans

**Figure 2:**
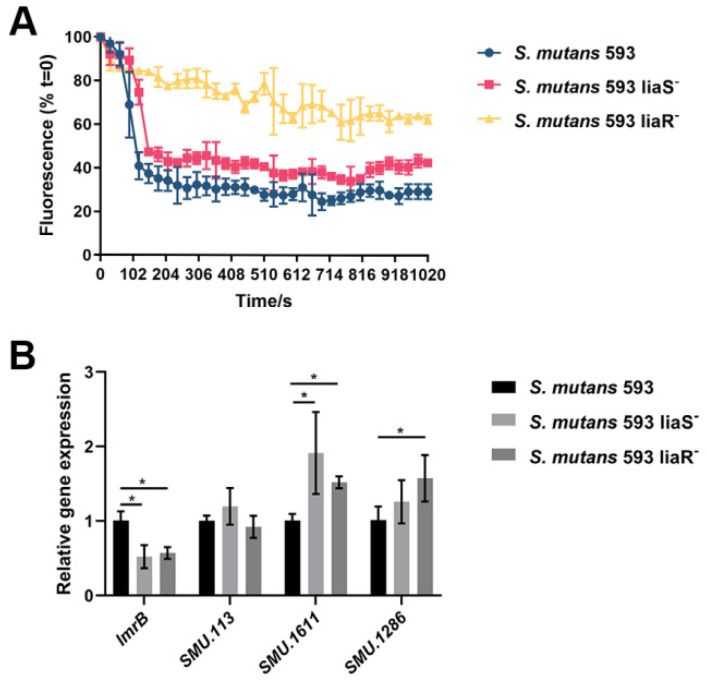
The efflux pump of *S. mutans*. (**A**) The efflux rates of *S. mutans*. (**B**) Expression levels of *lmrB*, *SMU. 113*, *SMU. 1611* and *SMU. 1286*. Values are presented as the mean ± SD. *, *p* < 0.05.

Figure 2A presents the efflux activity of *S. mutans* strains. Nile Red is an intracellular dye that is a common substrate for efflux pumps. The results showed that the rates of extrusion of the dye from the cells of the *liaS* and *liaR* deletion mutants were reduced, suggesting that the efflux pumps were suppressed. Figure 2B exhibited the expression of efflux-pump-encoding genes in *S. mutans* [27]. The results showed that the *lmrB* gene was downregulated in the two mutants when compared with the wild-type strain. SMU_1611c were upregulated in the two mutants when compared with the wild-type strain. The expression level of SMU_113c showed no significant difference in the mutants. The expression level of SMU_1286c in the *liaR* deletion mutant was higher than in the wild-type strain. While in the *liaS* deletion mutant, no significant difference was observed.

### 3.3. The Surface Charge of S. mutans

**Figure 3:**
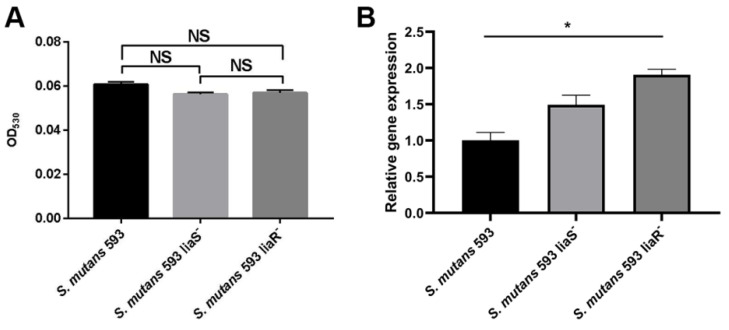
The surface charge of *S. mutans*. (**A**) The surface charge of *S. mutans*. (**B**) Expression level of *dltC*. Values are presented as the mean ± SD. *, *p* < 0.05. NS, *p* > 0.05.

Positively charged cytochrome c was used to assess the charge of the biofilm-grown cells based on the fact that it could bind to negatively charged cells [28]. The OD_530_ value represented the proportion of unbounded cytochrome c. The higher the value, the more positively charged the cell surface. As shown in Figure 3A, no significant difference was observed between the wild-type strain and the two mutants. The expression level of *dltC*, which might correlate with CHX resistance in *S. mutans*, was higher in the *liaR* deletion mutant. However, there was no significant difference in the expression of the *dltC* gene between the wild-type strain and *liaS* deletion mutant (Figure 3B).

### 3.4. The Ability of CESRs in S. mutans

**Figure 4:**
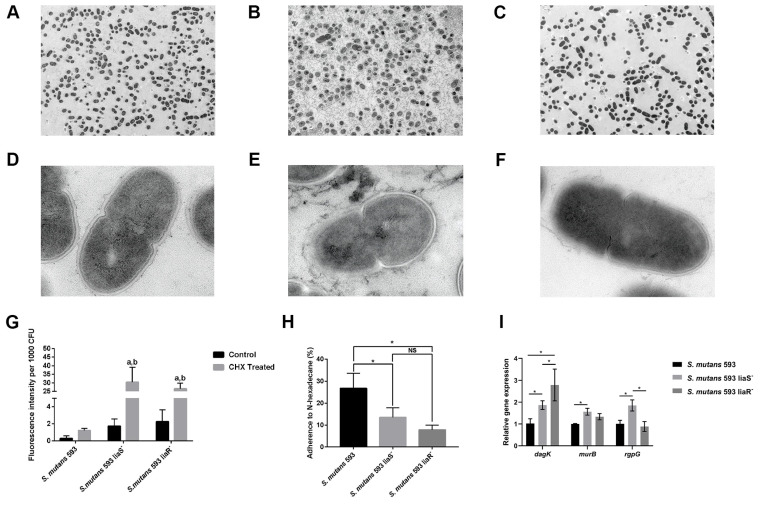
The CESRs of *S. mutans*. (**A**–**F**) TEM images showing the morphology of cells from *S. mutans* 593 ((**A**), ×2.5 k and (**D**), ×50 k), *S. mutans* 593 liaS^−^ ((**B**), ×2.5 k and (**E**), ×50 k) and *S. mutans* 593 liaR^−^ ((**C**), ×2.5 k and (**F**), ×50 k). (**G**) Cell surface hydrophobicity of *S. mutans*. (**H**) The percentages of positive cells stained with the DiBAC4(3) probe. (**I**) Expression levels of *dagK*, *murB* and *rgpG*. Values are presented as the mean ± SD. ^a^, significant differences at *p* < 0.05 compared to the control group; ^b^, significant differences at *p* < 0.05 compared to *S. mutans* 593; *, *p* < 0.05; NS, *p* > 0.05.

To detect the ability of CESRs in *S. mutans*, we first performed TEM to observe the changes in the membrane, as the cell membrane is essential for resistance to CHX. As shown in Figure 4A–F, the border of the cell membrane was unclear in the mutants compared with the wild-type strain.

The ability to depolarize the cell membrane and the hydrophobicity are the indicators of cell disruption [29]. To monitor changes in the membrane’s polarization, we first used DiBAC4(3), which is an anionic potential-response membrane probe. It can be excluded from healthy cells. In damaged cells whose membrane polarization is altered, the dye can enter the cells and bind to intracellular proteins and membranes, accompanied by increased fluorescence. Figure 4G shows the evidently increased intensity of fluorescence in the *liaS* and *liaR* deletion mutants compared with the wild-type strain with or without CHX treatment. All strains with CHX treatment exhibited stronger fluorescence intensity than the cells without CHX treatment.

In terms of hydrophobicity, the mutants showed significantly lower hydrophobicity when compared with the wild-type strain (Figure 4H). In summary, the higher depolarization and reduced hydrophobicity of the mutants indicated that the membrane of the *liaS* and *liaR* gene deletion mutants in *S. mutans* may be more easily disrupted.

The high level of depolarization and hydrophobicity implied that the cell membranes of the mutants were more likely to be disturbed. Therefore, we performed the RT-qPCR to analyze the expression levels of *dagK*, *murB* and *rgpD*, which are involved in membrane biosynthesis, remodeling and modification. Figure 4I shows that *dagK* was upregulated in the mutants. *murB* and *rgpD* were upregulated in the *liaS* deletion mutant. But in the *liaR* deletion mutant, the expression of *murB* and *rgpD* showed no significant difference compared with the wild-type strain.

## 4. Discussion

CHX is considered to be the gold standard for the prevention of dental caries. Previous studies have shown that the deletion of the LiaSR two-component system may lead to increased sensitivity to CHX [16]. Our study verified the fact again and investigated the underlying mechanisms. To our best knowledge, this is the first in vitro study to demonstrate the relationship between the LiaSR two-component system and CHX resistance.

To analyze the role of the LiaSR two-component system in *S. mutans* under CHX treatment, we performed an MIC assay and a viability assessment. The results exhibited that the *liaS* and *liaR* deleted mutants were more sensitive to CHX, which was in accordance with the study by Suntharalingam et al. [16]. The increased sensitivity of the mutants prompted us to explore the underlying mechanisms.

Multidrug efflux pumps are membrane proteins that contain multiple transmembrane domains that form channels to remove the CHX from the cytoplasm and reduce the intracellular CHX concentrations [30]. In *S. aureus* [31], *Pseudomonas aeruginosa* (*P. aeruginosa*) [32] and *Klebsiella pneumoniae* (*K. pneumoniae*) [33], the major facilitator superfamily (MFS) was one of the multidrug efflux pumps that contributed to CHX resistance. It had two domains that underwent a conformational switch between the inward-open and outward-open states while facilitating efflux activities [34]. On the basis of this, we speculated that the LiaSR two-component system could modulate the efflux of *S. mutans*, leading to a decrease in the accumulation of CHX, thereby facilitating the cells’ survival under CHX treatment. Our study showed that after deletion of the *liaS* and *liaR* genes, the efflux rates of *S. mutans* were significantly reduced. To explore the putative regulated efflux-pump-encoding genes, we analyzed the expression levels of *lmrB*, SMU_113c, SMU_1611c and SMU_1286c, which might be related to CHX resistance in *S. mutans* [27]. Consistently, the expression of *lmrB* was downregulated in the mutants, indicating that the LiaSR two-component system may regulate the *lmrB* gene to induce *S. mutans* to pump out CHX, which may reduce sensitivity to CHX. SMU_113c, SMU_1611c and SMU_1286c in the mutants showed upregulation or no significant difference compared with the wild-type strain. The results tied in well with a previous study [27]. In that study, SMU_113c, SMU_1611c and SMU_1286c were upregulated in the *lmrB* deletion mutant, accompanied by increases in exopolysaccharides and the extracellular matrix [27]. The increase in exopolysaccharides and the extracellular matrix could also be observed in the *liaS* and *liaR* deletion mutants [35], suggesting that this two-component system may regulate the *lmrB* gene indirectly.

Martin et al. [26] found that *S. mutans* could upregulate the expression of the *dlt* operon to increase the positive charge, which might contribute to the resistance to gentamicin. The bactericidal mechanism of CHX is similar to that of gentamicin. It can attach to the negatively charged cell’s surface due to its positive charge, leading to leakage of the cell’s components. A previous study has shown that the expression of *dltC* was upregulated in CHX-resistant *S. mutans* strains [9]. Given these findings, we speculated that the LiaSR two-component system may modulate the *dltC* gene to increase the positively charged cells’ surface and the ability to resist CHX in *S. mutans*. However, our results showed no significant difference in the cell surface charge of mutants compared with the wild-type strain. The expression of *dltC* was upregulated in the mutants. These findings did not correlate with an increased sensitivity to CHX in this study. On the basis of these findings, we speculated that the LiaSR two-component system did not enable the *dltC* gene to resist CHX.

The cell membrane is the outer barrier that is critical for viability in bacteria. CHX is a membrane-targeting antiseptic that can attach to and disrupt the cell membrane, resulting in the formation of ion channels, electrochemical degree destruction and membrane depolarization, which ultimately lead to bacterial death [36]. Therefore, we first carried out TEM observations. The images showed that although the integrity of the cell membrane in the mutants was maintained, the boundary was unclear in the mutants compared with the wild-type strain. The phenomenon may partly explain why the sensitivity of mutants to CHX increased.

The degree of bacterial membrane depolarization represents the state of membrane perturbation [29]. Our results showed that the mutants exhibited higher membrane depolarization after the CHX treatment. Hydrophobicity is another indicator of membrane disturbance [29]. A previous study confirmed that CHX-resistant *S. mutans* strains’ hydrophobicity was significantly elevated [9]. In this study, after deleting the *liaS* and *liaR* genes in *S. mutans*, the mutant strains’ hydrophobicity was reduced. This result was in accordance with the previous study [9]. Taken together, the membrane of the mutants was more likely to be disrupted, which was consistent with a previous study showing that the LiaSR two-component system participated in membrane homeostasis [22,37].

In *B. subtilis*, the LiaSR two-component system, which is homologous to *S. mutans*’ LiaSR two-component system, contributed to the cell envelope stress responses (CESRs) [38,39,40]. When bacterial cells sense a disturbance, the ability of the envelope and modification of the membrane will be activated to maintain homeostasis and repair the damage. Suntharalingam et al. found that after 10 min of bacitracin treatment, *dagK*, *rgpG* and *murB*, which are related to membrane biosynthesis, remodeling and modification in the *liaFSR* deletion mutant, were reduced [16]. To determine whether *dagK*, *rgpG* and *murB* were regulated by the LiaSR two-component system to affect CESRs in *S. mutans*, we performed RT-qPCR. However, our study was inconsistent with the study of Suntharalingam et al. [16]. The expression levels of *dagK*, *rgpG* and *murB* were elevated or not significantly different in mutants, which indicated that these genes were not the target genes for the LiaSR two-component system in CESRs. Previous studies have validated that the LiaSR two-component system exerts positive effects on the expression of *spxA2* to maintain the membrane’s homeostasis, which is also essential for CHX resistance [16,22,37]. Accordingly, we speculated that the LiaSR two-component system mediation of CHX resistance relied on the expression of the *spxA2* gene. However, further studies are needed to explore the other target genes regulated by this two-component system.

In conclusion, our study demonstrated that the LiaSR two-component system is involved in CHX resistance in *S. mutans*. This system could increase the *lmrB* gene, which plays an important role in detoxifying intracellular CHX. It is also required for CESRs, which contributes to the decreased sensitivity to CHX. This study provides valuable insights into the mechanisms of the LiaSR two-component system in CHX-resistant *S. mutans*. However, more detailed mechanistic studies must be performed to prove the relationship between the LiaSR two-component system and the *lmrB* gene, which may be beneficial for the development of the LiaSR two-component system or efflux inhibitors as potential adjunct methods to therapeutically intervene against biofilms. CESRs would be activated when bacteria encounter many types of stress, such as mechanical forces and antiseptics. The CESR-related genes regulated by the LiaSR two-component system must be further investigated, which may contribute to the development of CESR-targeting antiseptics.

## Figures and Tables

**Table 1:** Bacterial strains used in this study.

Strain or Plasmid	Relevant Characteristics	Source
*S. mutans* 593	Wild-type, serotype c	[21]
*S. mutans* 593 liaS^−^	Defined *liaS* deletion mutant	[22]
*S. mutans* 593 liaR^−^	Defined *liaR* deletion mutant	

**Table 2:** Primers used in this study.

Primers	Sequence (5′→3′)
*dltC*-F	ACCAAGGCTGTACTTAATCAGG
*dltC*-R	AGGATGCAGATAAAGGAAGCC
*dagK*-F	GGGATCCTTCTCATGTTCTCTC
*dagK*-R	CATCTGCATTGACTTCCATCATT
*murB*-F	GCAGTGCTAAGACTCCCGAATC
*murB*-R	TTGCGGAAGTGTGGAGATTGGC
*rgpB*-F	GGAATCGTTAGAAGCAAAAGGTG
*rgpB*-R	TGACCAAAGCAGTAACCCTC
*lmrB*-F	CGTCATCTCTTTATCGCAAGCATC
*lmrB*-R	CAGTATGTTCCAGCACTTCAGCC
SMU_113-F	AAATACGGTGACACTTGCTGGTAAG
SMU_113-R	TCAATAGATGGCACAACGGTAATC
SMU_1611-F	ACCGCTATGCTAAGGCTCTC
SMU_1611-R	AACCTATCTGAAGACGCTTGTC
SMU_1286-F	TGCCACAGGATGCAGAAGG
SMU_1286-R	TGTTGCACATCCTTACCCGT
16S-F	AGCGTTGTCCGGATTTATTG
16S-R	CTACGCATTTCACCGCTACA

## Data Availability

Data are contained within the article.
